# The Effects of Long-Term High-Temperature Aging on the Microstructural Evolution and Impact Fracture Behavior of Inconel 625 Superalloy

**DOI:** 10.3390/ma19101932

**Published:** 2026-05-08

**Authors:** Zhining Li, Kejian Li, Yao Wu, Zhipeng Cai, Qu Liu

**Affiliations:** 1 Department of Mechanical Engineering, Tsinghua University, Beijing 100084, China; lzn23@mails.tsinghua.edu.cn (Z.L.); kejianli@mail.tsinghua.edu.cn (K.L.); wuyao_1209@163.com (Y.W.); czpdme@mail.tsinghua.edu.cn (Z.C.); 2 State Key Laboratory of Clean and Efficient Turbomachinery Power Equipment, Tsinghua University, Beijing 100084, China

**Keywords:** Inconel 625 superalloy, long-term high-temperature aging, impact toughness, microstructural evolution

## Abstract

Inconel 625 is widely used in high-temperature structural components because of its excellent strength, toughness, and corrosion resistance. However, long-term exposure to elevated temperatures can induce precipitation of carbides, γ″ phase, and δ phase, leading to microstructural degradation and reduced mechanical reliability. Although precipitation evolution and tensile properties of aged Inconel 625 have been widely studied, the relationship between long-term precipitate evolution and impact fracture behavior remains insufficiently clarified. In this study, solution-treated Inconel 625 alloy was aged at 700 °C and 750 °C for up to 5000 h, with additional stress-assisted aging at 750 °C under 30 MPa and 51 MPa. Impact toughness, microhardness, fracture morphology, and precipitate evolution were systematically investigated. The results show that long-term aging significantly reduces impact toughness at both room and elevated temperatures, with a more pronounced reduction at room temperature. The room-temperature impact energy decreases from 314 J to approximately 10 J and stabilizes after 2000 h. Quantitative analysis shows that γ″ precipitate coarsening follows the Lifshitz–Slyozov–Wagner relationship, indicating diffusion-controlled growth. Stress-assisted aging under the present low stress levels has only a limited influence on precipitate evolution and impact toughness. The toughness degradation is mainly attributed to chain-like grain-boundary carbides and needle-like or plate-like δ phase, which embrittle grain boundaries, segment the austenitic matrix, and limit impact energy absorption.

## 1. Introduction

The solid-solution-strengthened nickel-based superalloy Inconel 625 has attracted considerable attention because of its excellent high-temperature strength, high toughness, and outstanding resistance to oxidation and corrosion. It is widely used in aerospace engines, marine systems, the petrochemical industry, and critical components of thermal and nuclear power plants, where long-term microstructural stability and mechanical reliability are required under elevated-temperature service conditions [1]. In addition, Inconel 625 exhibits good workability and excellent weldability, making it suitable for a wide range of manufacturing processes and high-temperature structural applications [2].

The main alloying elements of Inconel 625 include chromium (20–23 wt%), molybdenum (8–10 wt%), and niobium (3.15–4.15 wt%). Chromium and molybdenum are primarily dissolved in the nickel matrix and provide solid-solution strengthening, whereas niobium, together with minor additions of aluminum and titanium, can form precipitation-strengthening phases such as γ″ (Ni_3_Nb) and γ′ (Ni_3_Al/Ti). These precipitates contribute to the alloy’s high-temperature strength and creep resistance. However, during prolonged exposure to high temperatures, the type, amount, morphology, and distribution of precipitates can change substantially. Such microstructural evolution may alter the balance between strengthening and embrittlement, thereby affecting the long-term mechanical stability of the alloy.

Previous studies have shown that long-term aging of Inconel 625 and related GH625 alloys promotes the precipitation of grain-boundary carbides, intragranular γ″ phase, and δ phase, leading to significant changes in mechanical properties. Guo Yan et al. [3] reported that, after aging at 760 °C, M_23_C_6_ carbides precipitated along grain boundaries, while γ″ and γ′ phases formed within grains, resulting in a marked decrease in room-temperature impact toughness. They also found that after aging at 760 °C for 3000 h, the yield strength increased slightly, whereas the tensile strength showed no significant change [4]. Li Yamin et al. [5] investigated the precipitation evolution of domestically produced GH625 alloy during long-term aging at 720 °C and showed that aging promotes the transformation from γ to γ″, followed by δ phase, which improves yield and tensile strength but reduces ductility. Other studies [6,7] have also examined the microstructural evolution and mechanical-property changes of Alloy 625 during long-term aging.

Although these studies have clarified the general precipitation sequence and its influence on tensile properties or room-temperature toughness, several issues remain insufficiently understood. First, most existing work has focused on either a single aging temperature or relatively limited aging durations, whereas systematic data after prolonged aging beyond 3000 h remain limited. Second, the degradation of impact toughness has rarely been evaluated simultaneously at room and elevated testing temperatures. Third, the relationship between precipitate evolution, fracture morphology, and impact toughness degradation in domestically produced Inconel 625 alloy still requires further clarification. In particular, aging at 700 °C and 750 °C is of practical relevance because this temperature range is close to the long-term service conditions of high-temperature components and lies within the sensitive precipitation regime for grain-boundary carbides, γ″ phase, and δ phase. Comparing these two aging temperatures therefore helps clarify how precipitation kinetics affect impact fracture behavior and toughness degradation.

Therefore, this study systematically investigates the microstructural evolution, precipitation behavior, and impact fracture behavior of solution-treated Inconel 625 alloy after long-term aging at 700 °C and 750 °C for up to 5000 h. Stress-assisted aging at 750 °C under nominal stresses of 30 MPa and 51 MPa was also conducted to evaluate the influence of service-relevant low stress levels. By correlating impact energy, microhardness, fracture morphology, and precipitate evolution, this work aims to clarify the microstructural origin of toughness degradation in long-term aged Inconel 625 alloy and to provide guidance for reliability assessment under high-temperature service conditions.

## 2. Materials and Methods

### 2.1. Experimental Material

The experimental material used in this study was Inconel 625 alloy tubing (Special Metals Corporation, New Hartford, NY, USA). The tubes were produced by industrial-scale vacuum induction melting (VIM) followed by electroslag remelting (ESR). The resulting ingots were processed into billets, hot-extruded into hollow shells, and subsequently cold-rolled into final tubes. The chemical composition of the alloy is listed in Table 1.

### 2.2. Long-Term High-Temperature Aging Treatment

To ensure a uniform initial microstructure, specimens were longitudinally cut from the cold-rolled tubes, solution-treated at 1150 °C for 2 h, and then water-quenched. Long-term aging treatments were conducted at 700 °C and 750 °C for various durations. These temperatures were selected based on the typical service temperature and the maximum operating temperature of the material. The aging experiments were carried out using a high-temperature testing system equipped with a furnace. Stress-free aging was performed on cylindrical specimens with dimensions of Φ65 × 55 mm, whereas stress-assisted aging was conducted on cylindrical specimens with dimensions of Φ32 × 150 mm. During aging, the temperature deviation was controlled within ±3 °C, and the temperature gradient was maintained below 3 °C.

To evaluate the effect of stress-assisted aging on microstructural evolution, additional specimens were aged under nominal stresses of 30 MPa and 51 MPa. These stress levels were determined based on the actual service loading conditions of the material and the allowable stress specified in the ASME code. The detailed aging schedules are listed in Table 2.

After aging, impact tests were performed using standard Charpy V-notch specimens, with three specimens tested for each aging condition. The specimen dimensions were 10 mm × 10 mm × 55 mm, and the impact toughness was measured in accordance with GB/T 2650-2022 [8]. For high-temperature impact tests, the specimens were heated to 750 °C and immediately removed from the furnace for testing. Following the impact tests, Vickers hardness measurements were conducted. Five regions were randomly selected from each specimen, and ten indentations were made in each region. After excluding the upper and lower 15% of the measured values, the remaining data were averaged to obtain the final hardness value.

### 2.3. Microstructural Characterization

Metallographic specimens were prepared by sequential grinding using SiC papers with grit sizes of 400, 800, 1000, 1200, 1500, 2000, and 3000, followed by polishing with a W0.5 diamond suspension. After polishing, the specimens were ultrasonically cleaned successively in ethanol and acetone. They were then etched for 120 s using a solution composed of 100 mL ethanol, 100 mL HCl, and 5 g CuCl_2_ (Shanghai Acmec Biochemical Technology Co., Ltd., Shanghai, China).

The microstructures of Inconel 625 alloy aged for different durations were first examined using an Olympus DP72 optical microscope (OM) (Olympus Corporation, Tokyo, Japan) at relatively low magnifications. Detailed microstructural observations were then performed using a ZEISS GeminiSEM 300 scanning electron microscope (SEM) (Carl Zeiss AG, Oberkochen, Germany) at a working distance of approximately 10 mm. SEM imaging was conducted in secondary-electron mode, with magnifications ranging from 500× to 50,000×. An energy-dispersive X-ray spectroscopy (EDS) system (Carl Zeiss AG, Oberkochen, Germany) attached to the SEM was used to analyze the chemical compositions of selected microregions. In addition, the microhardness of the aged specimens was measured using an FM-810 digital automatic microhardness tester (Future-Tech Corp., Kawasaki, Japan) under a load of 200 gf and a dwell time of 10 s.

## 3. Results and Discussion

### 3.1. Impact Toughness

**Figure 1:**
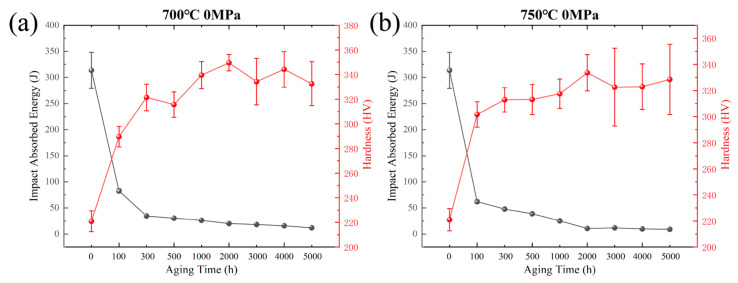
Evolution of room-temperature impact properties and microhardness of solution-treated Inconel 625 alloy with aging time after long-term aging at different temperatures: (**a**) 700 °C for 5000 h; (**b**) 750 °C for 5000 h.

Figure 1 shows the room-temperature impact energy and microhardness of solution-treated Inconel 625 alloy after long-term aging at 700 °C and 750 °C for different durations. The impact energy exhibits a similar decreasing trend at both aging temperatures. The solution-treated alloy shows a high room-temperature impact energy of approximately 314 J, indicating excellent toughness. After aging for 100 h, however, the impact energy decreases sharply to below 100 J, demonstrating severe degradation in impact toughness. With prolonged aging, the impact energy continues to decrease, but the rate of decline gradually slows. After aging for more than 2000 h at both 700 °C and 750 °C, the room-temperature impact energy stabilizes at approximately 10 J.

In contrast, the microhardness increases with aging time. The microhardness of the solution-treated alloy is approximately 210 HV, increases rapidly to about 300 HV after 100 h of aging, and then rises more slowly before stabilizing after 2000 h. The opposite trends in impact energy and microhardness suggest that precipitation during high-temperature aging strengthens the alloy but reduces its ability to absorb impact energy. This reduction in room-temperature impact toughness is therefore closely associated with precipitate formation both within grains and along grain boundaries.

**Figure 2:**
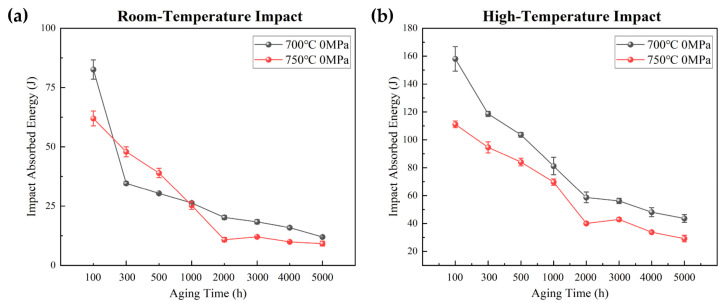
Effect of different aging temperatures on the room-temperature and high-temperature impact properties of Inconel 625: (**a**) room-temperature impact; (**b**) high-temperature impact (750 °C).

Figure 2 illustrates the effect of aging temperature on the room-temperature and high-temperature impact toughness of Inconel 625 alloy. As shown in Figure 2a, the room-temperature impact energy decreases more sharply after aging at 750 °C than after aging at 700 °C, especially within the first 100 h. With further increase in aging time, the impact energy gradually decreases and stabilizes after approximately 2000 h. In contrast, the specimens aged at 700 °C show a more gradual decrease in room-temperature impact energy. After 5000 h of aging, the impact energy of specimens aged at both temperatures tends to converge to similar values. The high-temperature impact results in Figure 2b show a similar trend, with a more pronounced reduction in impact energy for specimens aged at 750 °C. These results indicate that aging at higher temperatures leads to more significant precipitation behavior and exert a stronger influence on the mechanical properties of the solution-treated alloy.

**Figure 3:**
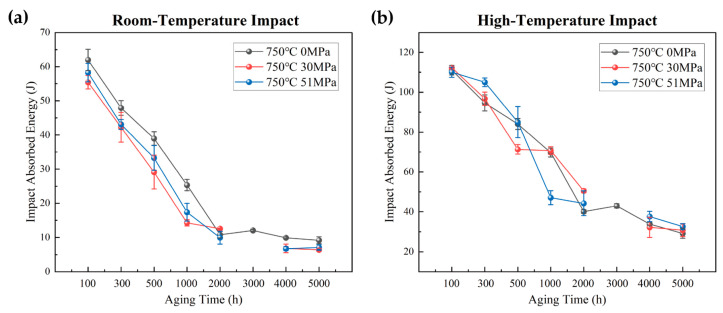
Effect of different applied stresses (0, 30, and 51 MPa) during aging on the room-temperature and high-temperature impact properties of Inconel 625 alloy: (**a**) room-temperature impact; (**b**) high-temperature impact at 750 °C.

Figure 3 shows the effect of applied stresses during long-term aging at 750 °C on the room-temperature and high-temperature impact toughness of Inconel 625 alloy. The applied stresses were 0, 30, and 51 MPa. The results indicate that applied stress causes only a slight reduction in both room-temperature and high-temperature impact toughness. However, this reduction is limited, and the variation in impact toughness with aging time remains essentially similar under all stress conditions. This suggests that stress-assisted aging does not markedly alter the precipitate evolution of the alloy. The limited effect of applied stress may be attributed to the fact that the applied stress levels are considerably lower than the high-temperature strength of Inconel 625 alloy at 750 °C.

**Figure 4:**
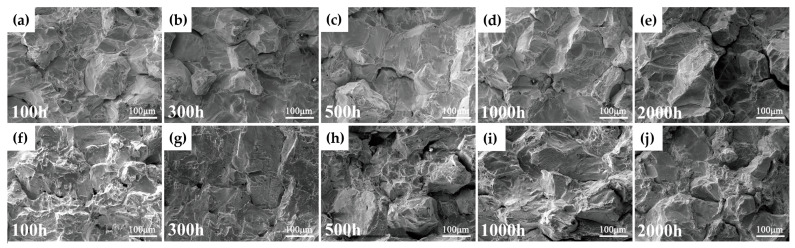
Fracture morphologies (SEM) of Inconel 625 alloy after aging at 750 °C for different durations: (**a**–**e**) room-temperature impact fracture surfaces with aging times of 100–2000 h; (**f**–**j**) high-temperature (750 °C) impact fracture surfaces with aging times of 100–2000 h.

Figure 4 shows the fracture morphologies of Inconel 625 alloy after aging at 750 °C for different durations under both room-temperature and high-temperature impact conditions. After aging for more than 100 h, the room-temperature fracture surfaces exhibit a typical “rock candy”-like brittle morphology, characteristic of intergranular fracture, with very few dimples and almost no plastic deformation. This indicates a rapid ductile-to-brittle transition under long-term aging at 750 °C, consistent with the previously observed decrease in room-temperature impact energy. These intergranular fracture features also suggest that grain boundary precipitation during aging plays a critical role in the degradation of impact toughness.

For specimens aged at 750 °C for 100 h and tested at 750 °C, the intergranular fracture features are less pronounced. The fracture surfaces exhibit numerous shallow dimples and dimple bands, indicating a certain degree of ductile fracture behavior, which can be attributed to the improved plasticity of the matrix at elevated temperatures. With increasing aging time, the number of shallow dimples gradually decreases, while cleavage facets become more prominent, indicating a progressive reduction in high-temperature toughness. After aging for 1000 h, the fracture surfaces also exhibit a “rock candy”-like intergranular brittle morphology. Moreover, the difference in impact toughness between room temperature and 750 °C gradually decreases with increasing aging time.

### 3.2. Microstructural Analysis

**Figure 5:**
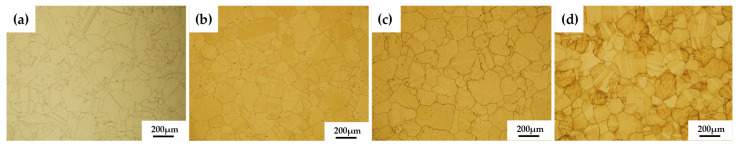
Optical microstructures of solution-treated Inconel 625 alloy after aging at 750 °C for different durations: (**a**) solution-treated state; (**b**) aged for 100 h; (**c**) aged for 300 h; (**d**) aged for 2000 h.

Figure 5 presents the optical microstructures of solution-treated Inconel 625 alloy after aging at 750 °C for different durations. During long-term high-temperature aging, the grain size remains essentially unchanged, with a relatively uniform distribution in the range of 100–140 μm. The solution-treated alloy consists of an equiaxed austenitic matrix with a small number of randomly distributed primary carbide particles and some annealing twins within the grains. After aging at 750 °C for 100 h, fine chain-like carbide particles precipitate along the grain boundaries, making the grain boundaries more clearly visible after etching. With prolonged aging, an increased density of fine intragranular precipitates is observed, leading to enhanced etching contrast. When the aging time exceeds 500 h, needle-like phases appear at the grain boundaries and gradually extend into the grain interiors. The microstructural evolution of solution-treated Inconel 625 alloy during aging at 700 °C follows a similar trend, but the precipitation kinetics are slower because of the lower aging temperature. The detailed differences in microstructural evolution are further analyzed using scanning electron microscopy.

**Figure 6:**
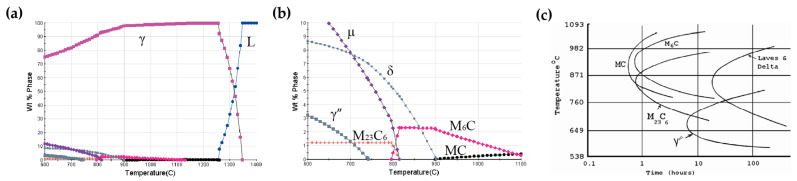
Precipitated phases and isothermal transformation diagram of Inconel 625 alloy: (**a**) calculated equilibrium phase diagram; (**b**) enlarged view of (**a**); (**c**) time–temperature–transformation (TTT) diagram [9].

Based on the chemical composition of Inconel 625 alloy, thermodynamic simulations of precipitated phases were performed using JMatPro software(Version 7.0), as shown in Figure 6a,b. The calculated phases include the γ matrix, γ″ phase, δ phase, various carbides, and minor topologically close-packed (TCP) phases. Among these phases, MC-type carbides precipitate directly from the liquid phase and are randomly distributed at grain boundaries or within grains. Because of their low volume fraction, they have only a limited effect on the mechanical properties of the alloy. As the temperature decreases, M_6_C and M_23_C_6_ carbides gradually precipitate, mainly along grain boundaries. These carbides can pin grain boundaries and inhibit grain boundary migration, thereby improving the high-temperature creep resistance of the alloy. With a further decrease in temperature, γ″ and δ phases begin to precipitate. Fine disk-shaped γ″ precipitates form within the grains and provide significant precipitation strengthening. However, γ″ is a metastable phase that tends to coarsen and transform into δ phase during prolonged high-temperature exposure.

In addition, the time–temperature–transformation (TTT) diagram of Inconel 625 alloy shown in Figure 6c [9] indicates that the primary precipitates during long-term aging at 700 °C and 750 °C are γ″, δ phases, and carbides. Although γ″ and δ phases share the same composition (Ni_3_Nb), they differ in crystal structure. During long-term aging at 750 °C, M_23_C_6_ carbides are expected to precipitate first, followed by the formation of fine γ″ precipitates, which subsequently transforms into the δ phase with increasing aging time.

**Figure 7:**
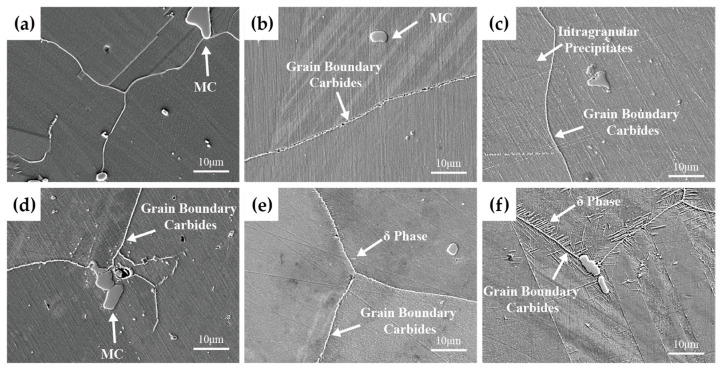
Microstructural morphologies (SEM) of solution-treated Inconel 625 alloy after aging at 700 °C for different durations: (**a**) solution-treated state; (**b**) aged for 100 h; (**c**) aged for 300 h; (**d**) aged for 500 h; (**e**) aged for 1000 h; (**f**) aged for 2000 h.

Figure 7a shows the microstructure of solution-treated Inconel 625 alloy. A small number of coarse primary particles are randomly distributed both along grain boundaries and within the grains. Based on descriptions in the relevant literature, these particles are considered to be MC carbides [10]. Figure 7b presents the microstructure after aging at 700 °C for 100 h. Fine particulate precipitates can be clearly observed along the grain boundaries. According to the TTT diagram [9] of Inconel 625 alloy and the relevant literature [11], these precipitates are considered to be M_23_C_6_ carbides. When the aging time increases over 300 h (Figure 7c,d), the amount of grain boundary carbides increases and forms a chain-like distribution. Meanwhile, the intragranular precipitate number density increases significantly, identified as γ″ precipitates. Consistent with the impact results described above, the impact toughness at this stage decreases significantly compared with that of the solution-treated alloy, whereas the microhardness increases markedly. Figure 7e shows the microstructure after aging at 700 °C for 1000 h, where needle-like δ phase begins to precipitate. These precipitates are mainly distributed along grain boundaries, exhibiting preferential orientation relationships. With further aging to 2000 h (Figure 7f), the needle-like phase grows rapidly along grain boundaries and twin boundaries and is inferred, based on the relevant literature [12], to be the δ phase, and extends progressively into the grain interior. At this stage, the impact toughness of Inconel 625 alloy gradually decreases with the growth and coarsening of grain boundary carbides and needle-like δ phase.

**Figure 8:**
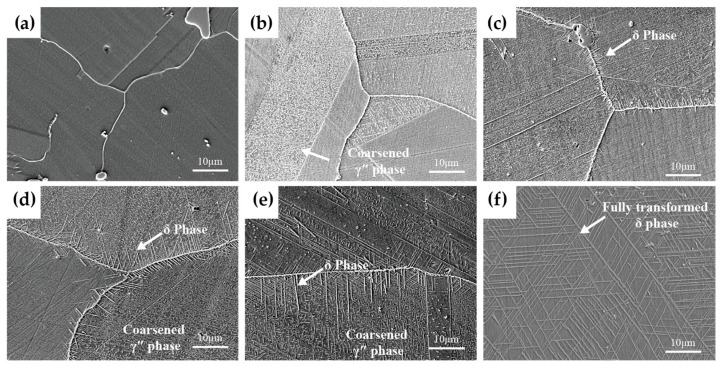
Microstructural morphologies (SEM) of solution-treated Inconel 625 alloy after aging at 750 °C for different durations: (**a**) solution-treated state; (**b**) aged for 100 h; (**c**) aged for 300 h; (**d**) aged for 500 h; (**e**) aged for 1000 h; (**f**) aged for 2000 h.

Figure 8 presents the microstructures of solution-treated Inconel 625 alloy after long-term aging at 750 °C. Figure 8a–f correspond to aging times of 0 h, 100 h, 300 h, 500 h, 1000 h, and 2000 h, respectively. Compared with aging at 700 °C, the higher aging temperature significantly accelerates the microstructural evolution of the alloy. As shown in Figure 8b, after aging at 750 °C for 100 h, a high density of chain-like carbides is observed along grain boundaries, accompanied by numerous fine γ″ precipitates within the grains. After aging for 300 h, the precipitation of needle-like δ phase along grain boundaries becomes pronounced. With further aging to 2000 h, carbides continue to precipitate and coarsen along grain boundaries in a chain-like morphology. More notably, the needle-like δ phase nucleates at grain boundaries and twin boundaries, precipitates along specific orientations, and grows into the grain interior in an intersecting manner, eventually forming Widmanstätten-type δ phase that spreads throughout the grains, as shown in Figure 8f. At this stage, the impact toughness of Inconel 625 alloy reaches its minimum level and stabilizes, showing no further significant change with increasing aging time.

**Figure 9:**
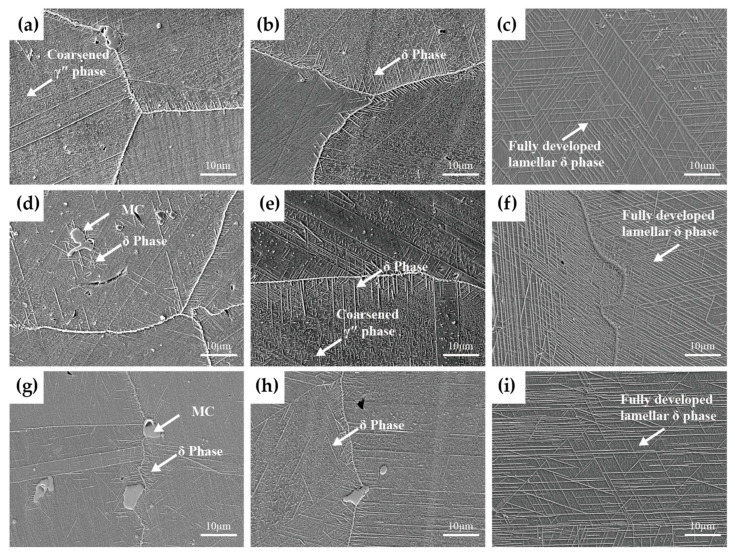
Effect of applied stress on the microstructural evolution of solution-treated Inconel 625 alloy during long-term aging at 750 °C (SEM): (**a**) 300 h without applied stress; (**b**) 500 h without applied stress; (**c**) 2000 h without applied stress; (**d**) 300 h under 30 MPa; (**e**) 500 h under 30 MPa; (**f**) 2000 h under 30 MPa; (**g**) 300 h under 51 MPa; (**h**) 500 h under 51 MPa; (**i**) 2000 h under 51 MPa.

Figure 9 illustrates the effect of applied stress on the microstructural evolution of solution-treated Inconel 625 alloy during long-term aging at 750 °C. Compared with the aging temperature, the applied stresses of 30 MPa and 51 MPa have only a limited influence on the precipitation rate of secondary phases. This can be attributed to the fact that precipitation in the alloy is a thermally activated diffusion-controlled process. Although the applied stress may slightly promote atomic diffusion, this suggests that the relatively low stress levels used in this study result in temperature remain the dominant factor governing diffusion and precipitation behavior.

### 3.3. Analysis and Discussion

Based on the microstructural evolution and impact toughness results of solution-treated Inconel 625 alloy after long-term aging at 700 °C and 750 °C, the significant reduction in impact toughness is closely associated with the continuous formation and growth of precipitates both along grain boundaries and within grains. Since the primary precipitates formed during high-temperature aging are carbides, γ″ phase, and needle-like δ phase, the following discussion focuses on the effects of these three types of precipitates on alloy performance.

**Figure 10:**
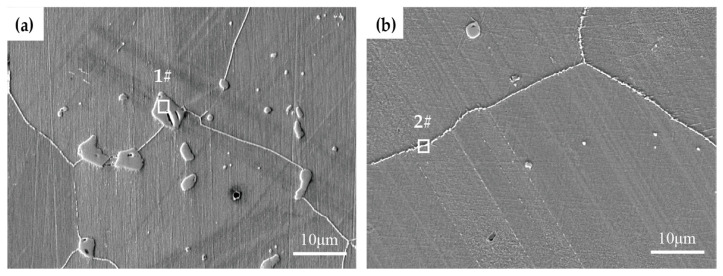
Carbide morphologies of Inconel 625 alloy after aging at 750 °C for 100 h (SEM&EDS): (**a**) particulate primary carbides; (**b**) grain boundary carbides.

Figure 10 shows the microstructure of solution-treated Inconel 625 alloy after aging at 750 °C for 100 h. The coarse particulate precipitates observed in Figure 10a were identified by EDS analysis (Table 3) as primary MC carbides rich in Nb and C. These carbides form during solidification and remain essentially unchanged in size and morphology during subsequent solution treatment and aging. Because of their low volume fraction, they are considered to have a negligible effect on the impact toughness of the alloy. In contrast, fine chain-like precipitates are observed along the grain boundaries. EDS analysis indicates that these precipitates mainly consist of Cr-rich M_23_C_6_ and Mo-rich M_6_C carbides. These carbides precipitate primarily during high-temperature aging. Although grain boundary carbides can inhibit grain boundary sliding and improve high-temperature creep strength, their extensive precipitation promotes grain boundary embrittlement [13]. This embrittlement is likely responsible for the early-stage decrease in impact toughness of solution-treated Inconel 625 alloy during high-temperature aging.

**Figure 11:**
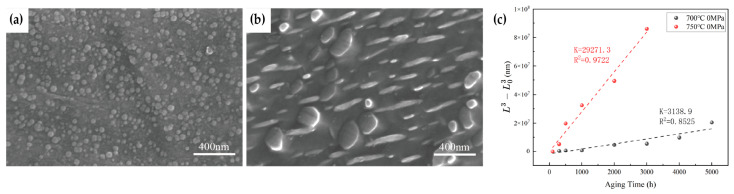
Morphology of intragranular γ″ precipitates in Inconel 625 alloy after aging at 750 °C for different durations (SEM): (**a**) 100 h; (**b**) 2000 h; (**c**) relationship between γ″ precipitate size and aging time.

In addition, the precipitation and growth behavior of the intragranular γ″ phase were examined using high-magnification SEM, as shown in Figure 11. After aging at 750 °C for 100 h, in addition to chain-like carbides along grain boundaries, numerous fine γ″ precipitates are observed within the grains. These precipitates are densely distributed in a near-spherical morphology, with an average particle size of approximately 46 nm. After prolonged aging for 2000 h, the fine γ″ particles gradually coarsen, merge, and grow, with the average particle size increasing to about 168 nm. Their distribution becomes sparser, and their morphology gradually evolves from spherical to disk-shaped. Using ImageJ (Version 1.54k) image analysis, the size of intragranular γ″ precipitates under different aging times at 700 °C and 750 °C was quantitatively evaluated. Previous studies [14] have shown that the precipitation of γ′ and γ″ phases in nickel-based alloys is closely related to elemental diffusion and follows the Lifshitz–Slyozov–Wagner (LSW) diffusion-controlled theory. The present results further indicate that the coarsening behavior of the γ″ phase conforms to the LSW model, as shown in Figure 11, in which the cube of the particle size varies linearly with aging time: $L^{3}-L_{0}^{3}=Kt$, where $L_{0}$ is the initial particle size and K is the coarsening rate constant. For the specimens aged at 700 °C under 0 MPa, the coarsening rate constant *K* obtained by fitting to the LSW model is 3138.9 nm^3^/h, with an $R^{2}$ of 0.8525. For the specimens aged at 750 °C under 0 MPa, the fitted K value is 29,271.3 nm^3^/h, with an $R^{2}$ of 0.9722. By comparison, the K value at 750 °C is significantly higher than that at 700 °C—approximately 9.3 times greater—indicating that temperature has a pronounced effect on the coarsening rate of the γ″ phase. Therefore, service temperature must be carefully considered when predicting the lifetime of related components. According to the literature [14], this behavior can be attributed to the diffusion-controlled nature of the coarsening process, which is governed by the diffusion activation energy. At higher temperatures, atomic diffusion is significantly enhanced. In Inconel 625 alloy, the coarsening of γ″ precipitates is primarily controlled by the diffusion of Nb atoms.

Finally, with further aging, needle-like δ phase precipitates along grain boundaries and twin boundaries. The δ phase grows and coarsens into the grain interior along preferred orientations, gradually forming plate-like features throughout the grains and eventually producing a Widmanstätten-type δ structure. Previous studies [15] have shown that the precipitation of δ phase consumes the surrounding γ″ phase, leading to the formation of precipitate-depleted zones and a reduction in grain boundary strength, thereby adversely affecting the impact toughness of the alloy.

**Figure 12:**
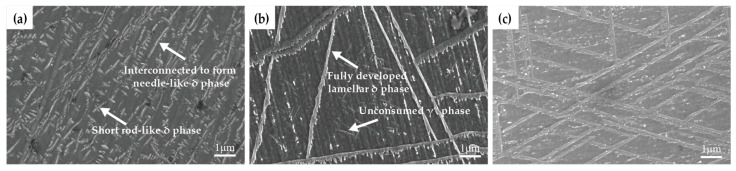
Transformation of intragranular γ″ phase into δ phase in Inconel 625 alloy after aging at 750 °C for different durations (SEM): (**a**) 300 h; (**b**) 2000 h; (**c**) 5000 h.

Figure 12 illustrates the transformation of intragranular γ″ phase into δ phase during aging at 750 °C. After aging for 300 h, some γ″ precipitates within the grains align in clusters along specific directions, while short rod-like δ phases precipitate perpendicular to the γ″ phase. As aging proceeds, the δ phase continues to nucleate and grow by consuming the γ″ phase, eventually linking together to form long needle-like δ precipitates. With further aging, the δ phase progressively absorbs surrounding γ″ precipitates and extends from grain boundaries into the grain interior. After aging at 750 °C for 2000 h, the δ phase spreads throughout the grains, and after 5000 h, γ″ phase is no longer observed. The long, plate-like δ phase together with a small amount of TCP phases, severely segment the austenitic matrix, hindering dislocation motion, and significantly reducing the ductility and toughness of the alloy. With further aging, δ phase precipitation gradually reaches saturation, and its effect on alloy properties becomes stable.

**Figure 13:**
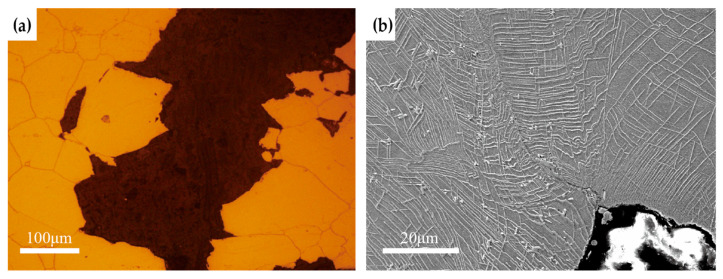
Cross-sectional morphology of the impact fracture of Inconel 625 alloy after aging at 750 °C for 2000 h: (**a**) cross-sectional morphology of the fracture surface (OM); (**b**) cross-sectional morphology of the fracture surface (SEM).

Figure 13 shows the cross-sectional morphology of the impact fracture of Inconel 625 alloy after aging at 750 °C for 2000 h. As shown in Figure 13a, the alloy predominantly undergoes intergranular fracture under impact loading after long-term aging. Only a small number of grains near the fracture surface exhibit very limited plastic deformation, which is insufficient to effectively absorb impact energy, thereby resulting in low impact toughness. As seen in Figure 13b, the grain interior is densely populated with plate-like δ phases. Due to their relatively high strength and brittleness compared with the matrix, these δ phases penetrate and segment the grains, effectively hindering plastic deformation via dislocation slip. Near the crack tip, only a narrow region containing δ phase, approximately 10 μm in width, undergoes slight bending deformation. Consequently, the energy absorbed under impact loading is limited, leading to rapid intergranular fracture. In summary, the extensive precipitation of chain-like carbides along grain boundaries and needle-like or plate-like δ phases is the primary factor responsible for the degradation of impact toughness in Inconel 625 alloy during long-term high-temperature aging.

## 4. Conclusions

During long-term aging of solution-treated Inconel 625 alloy at 700 °C and 750 °C, the grain size remains essentially stable at 100–140 μm, with no significant grain growth. Cr-rich M_23_C_6_ and Mo-rich M_6_C carbides preferentially precipitate along grain boundaries in a chain-like morphology, while numerous fine disk-shaped γ″ precipitates form within the grains. With increasing aging time, needle-like δ phase preferentially precipitates along grain boundaries and twin boundaries with preferred orientations. Meanwhile, intragranular γ″ phase gradually coarsens, merges, and transforms into Ni_3_Nb-type δ phase. Eventually, plate-like δ phase spreads throughout the grains, forming a Widmanstätten-type structure, and the microstructural evolution reaches a stable state.

During long-term aging at 700 °C and 750 °C, both room-temperature and high-temperature impact toughness decrease significantly with increasing aging time, with a more pronounced reduction at room temperature. When the aging time exceeds 2000 h, the impact toughness at both temperatures tends to stabilize. After aging, the room-temperature impact fracture is dominated by brittle intergranular fracture, indicating significant grain boundary embrittlement. At high temperature, the fracture mode is initially ductile in the early stages of aging but gradually transitions to intergranular brittle fracture after aging beyond 2000 h.

The microhardness of solution-treated Inconel 625 alloy increases significantly with increasing aging time at both 700 °C and 750 °C, showing a clear inverse correlation with impact toughness. This indicates that microstructural evolution and precipitation behavior during aging play a critical role in impact toughness degradation. In particular, the extensive precipitation of chain-like carbides along grain boundaries and needle-like δ phase is the primary cause of toughness degradation. These precipitates induce grain-boundary embrittlement and segment the grains, thereby hindering plastic deformation through dislocation slip, limiting impact energy absorption, and promoting rapid intergranular fracture.

## Figures and Tables

**Table 1:** Main chemical composition of Inconel 625 alloy (wt%).

C	Cr	Mo	Mn	Fe	Si	Ti	Nb	S	P	Co	Al	Cu	Ni
0.063	21.41	9.14	0.097	≤2.93	≤0.029	≤0.290	3.55	≤0.001	≤0.002	<0.002	0.300	<0.002	Bal.

**Table 2:** High-temperature aging schedules for Inconel 625 alloy.

No.	Temperature (°C)	Stress (MPa)	Aging Time (h)
1	700	0	0/100/300/500/1000/2000/3000/4000/5000
2	750	0	0/100/300/500/1000/2000/3000/4000/5000
3	750	30	0/100/300/500/1000/2000/3000/4000/5000
4	750	51	0/100/300/500/1000/2000/3000/4000/5000

**Table 3:** EDS analysis results of carbide composition in Inconel 625 alloy.

	C	Al	Si	Ti	Cr	Fe	Ni	Nb	Mo
1#	14.95	0.03	0.04	2.66	0.48	0.14	1.38	76.82	3.51
2#	8.13	0.31	0.05	0.28	27.65	2.89	46.47	3.56	10.68

## Data Availability

The original contributions presented in this study are included in the article. Further inquiries can be directed to the corresponding author.
